# CPT1A‐mediated succinylation of S100A10 increases human gastric cancer invasion

**DOI:** 10.1111/jcmm.13920

**Published:** 2018-11-05

**Authors:** Chao Wang, Chen Zhang, Xiang Li, Jiajia Shen, Yue Xu, Hui Shi, Xianmin Mu, Jinshun Pan, Ting Zhao, Mengjing Li, Biao Geng, Che Xu, Hao Wen, Qiang You

**Affiliations:** ^1^ Department of Surgery Second Affiliated Hospital Nanjing Medical University Nanjing Jiangsu China; ^2^ Department of Biotherapy Second Affiliated Hospital Nanjing Medical University Nanjing Jiangsu China; ^3^ Department of Surgery First Affiliated Hospital of Nanjing Medical University Nanjing Jiangsu China; ^4^ Department of Thoracic Surgery Affiliated Hospital of Nantong University Nantong Jiangsu China; ^5^ Medical Center for Digestive Diseases Second Affiliated Hospital Nanjing Medical University Nanjing Jiangsu China; ^6^ Key Laboratory for Aging & Disease Nanjing Medical University Nanjing Jiangsu China

**Keywords:** CPT1A, gastric cancer, metastasis, S100A10, SIRT5, succinylation, ubiquitylation

## Abstract

Gastric cancer (GC) is a malignancy of the lining of the stomach and is prone to distant metastasis, which involves a variety of complex molecules. The S100 proteins are a family of calcium‐binding cytosolic proteins that possess a wide range of intracellular and extracellular functions and play pivotal roles in the invasion and migration of tumour cells. Among these, S100A10 is known to be overexpressed in GC. Lysine succinylation, a recently identified form of protein post‐translational modification, is an important regulator of cellular processes. Here, we demonstrated that S100A10 was succinylated at lysine residue 47 (K47), and levels of succinylated S100A10 were increased in human GC. Moreover, K47 succinylation of S100A10 was stabilized by suppression of ubiquitylation and subsequent proteasomal degradation. Furthermore, carnitine palmitoyltransferase 1A (CPT1A) was found to function as a lysine succinyltransferase that interacts with S100A10. Succinylation of S100A10 is regulated by CPT1A, while desuccinylation is regulated by SIRT5. Overexpression of a succinylation mimetic mutant, K47E S100A10, increased cell invasion and migration. Taken together, this study reveals a novel mechanism of S100A10 accumulation mediated by succinylation in GC, which promotes GC progression and is regulated by the succinyltransferase CPT1A and SIRT5‐mediated desuccinylation.

## 1 INTRODUCTION

Gastric cancer (GC) is the fourth most common malignancy and the third leading cause of cancer mortality worldwide.^1^ In 2015, GC exhibited the second highest incidence of all cancers and was the second leading cause of cancer deaths in China.^2^ Many patients are diagnosed with advanced disease and present with recurrent disease after surgery. GC is prone to lymph node metastasis and exhibits a strong invasive ability.^3^ Thus, the identification of effective specific markers for the diagnosis and treatment of GC is critical.

S100 proteins contain at least one EF‐hand Ca^2+^‐binding motif and exhibit a broad range of intracellular and extracellular functions by regulating calcium homoeostasis, apoptosis, proliferation, differentiation, invasion, motility, cytoskeletal interactions, energy metabolism, and inflammation, all of which have been implicated in multiple stages of tumourigenesis and progression.^4^ As a member of the S100 protein family, S100A10, also known as annexin light chain or p11, contains two EF‐hand motifs.^5^ S100A10 binds to its annexin A2 ligand to form a heterotetrameric (S100A10)_2_(annexin A2)_2_ complex on various tumour cell membranes.^6^, ^7^, ^8^ This extracellular complex acts as a receptor for plasminogen and regulates plasminogen activator‐dependent plasminogen activation on cancer cells. Notably, the overexpression of S100A10 is related to tumour metastasis, invasiveness, and angiogenesis.^9^, ^10^, ^11^, ^12^, ^13^ While S100A10 is known to be overexpressed in GC, the molecular mechanisms underlying its effect on GC cell function and its regulation remain to be fully elucidated.^14^

Protein post‐translational modifications (PTMs) can alter the properties of proteins, such as their activity, cellular localization, stability, and interactions with other proteins, which can in turn influence cellular physiology.^15^, ^16^ Recently, many novel PTMs have been identified. Protein lysine residues can be subjected to various PTMs, including acetylation, propionylation, methylation, butyrylation, succinylation, crotonylation, malonylation, glutarylation, long‐chain fatty acylation, ubiquitylation and 2‐hydroxysobuturylation.^17^, ^18^, ^19^, ^20^, ^21^, ^22^, ^23^, ^24^ Previously, we demonstrated that succinate acts as an oncometabolite and functions as an initiator in tumourigenesis and tumour progression.^25^, ^26^ Moreover, protein lysine succinylation has been identified as a frequently occurring PTM^19^, ^27^ that plays an important role in regulating heart metabolism and cardiac function.^28^ Succinylation modifications are regulated by sirtuin (silent mating type information regulation 2 homologue, SIRT) 5, which removes succinyl modifications from lysine residues.^17^ Recently, lysine acetyltransferase 2A (KAT2A) and carnitine palmitoyltransferase 1A (CPT1A) were shown to have lysine succinyltransferase activities.^29^, ^30^

In this study, we examined the expression of S100A10 in GC and determined the succinylation of S100A10 via mass spectrometry. The proteins responsible for modifying (adding or removing) succinyl on S100A10 were further investigated, and the expression of CPT1A and SIRT5 in GC was also assessed. Moreover, the role of wild‐type (WT) or mutant S100A10 in GC progression was explored. To our knowledge, this is the first evaluation of the mechanism by which lysine succinylation contributes to the progression of GC.

## 2 MATERIALS AND METHODS

### 2.1 GC sample collection and preparation

Human tissue samples were obtained from seven patients diagnosed with GC who underwent surgical resection at the Second Affiliated Hospital of Nanjing Medical University (Jiangsu, China). Matching adjacent non‐tumour tissues were obtained from the part of the resected specimen furthest away from the tumour. The samples were washed with ice‐cold phosphate‐buffered saline (GE Healthcare, Beijing, China) to remove residual blood after surgical resection and then immediately snap‐frozen in liquid nitrogen and stored at −80°C for further analyses. No preoperative radiotherapy, chemotherapy, or other therapy was performed on the GC patients. This study was conducted according to the principles of the Declaration of Helsinki and was approved by the Hospital Ethics Committee of the Second Affiliated Hospital of Nanjing Medical University.

### 2.2 Animals

We utilized 6‐ to 8‐week‐old male WT C57BL/6J mice (Nanjing Biomedical Research Institute of Nanjing University). Mice were housed in a temperature‐controlled environment with a 12‐hour light‐dark cycle and were allowed free access to water and food. All animal procedures were approved by the Laboratory Animal Core Facility of Nanjing Medical University.

### 2.3 Cell culture and treatment

AGS (#ATCC^®^ CRL‐1739™) cells were purchased from Shanghai Cafa Biological Technology Co. Ltd (Shanghai, China). MGC‐803 (#TCHu84), SGC7901 (#TCHu46), 293T (Cat. #GNHu17), and B16‐F10 (#TCM36) cells were obtained from the Chinese Academy of Sciences (Shanghai, China). Cells were cultured in Dulbecco's modified Eagle's medium supplemented with 10% foetal bovine serum, streptomycin (100 μg/mL), and penicillin (100 U/mL). S100A10 levels were assessed in cells were treated with or without 10 mM MG132 (#S2619; Selleck, Shanghai, China) or 10 μg/mL cycloheximide (CHX, #HY‐12320; Selleck).

### 2.4 RNA isolation and qPCR

Total RNA was isolated from human tissues using Trizol reagent (Invitrogen, Carlsbad, CA, USA), following the manufacturer's instructions. The RNA was then analysed using real‐time qPCR with SYBR Green PCR Master mix (Roche Applied Science, Mannheim, Germany) on a StepOnePlus™ Real‐Time PCR System (Applied Biosystems, Foster City, CA, USA). The relative gene expression was normalized to GAPDH. Specific primer sets used for this assay included S100A10 (forward: AACAA AGGAG GACCT GAGAG TAC, reverse: CTTTG CCATC TCTAC ACTGG TCC), and GAPDH (forward: TTGCC ATCAA TGACC CCTTC A, reverse: CGCCC CACTT GATTT TGGA).

### 2.5 Western blotting

Total protein was prepared from gastric tissues or cultured cell samples using RIPA lysis buffer containing protease inhibitor cocktail (Roche Applied Science, Penzberg, Germany) and centrifuged at 10 000 *g* at 4°C for 15 minutes. Supernatants were mixed with SDS‐PAGE sample‐loading buffer, boiled for 5 minutes, and then subjected to SDS‐PAGE. After being transferred onto polyvinylidene fluoride membranes, non‐specific binding was blocked with 5% nonfat milk. The blots were probed with the following primary antibodies: S100A10 antibody (#5529; Cell Signaling, Danvers, MA, USA), rat monoclonal anti‐HA antibody (clone 3F10, #11867423001; Roche, Mannheim, Germany), mouse monoclonal ANTI‐FLAG^®^ M2 antibody (#F1804; Sigma‐Aldrich, St. Louis, MO, USA), succinyl lysine antibody (#PTM‐401; PTM Bio, Hangzhou, China), malonyl lysine antibody (#PTM‐901; PTM Bio), glutaryl lysine antibody (#PTM‐1151; PTM Bio), SIRT5 antibody (#8782; Cell Signaling Technology), human CPT1A antibody (#12252; Cell Signaling Technology), mouse CPT1A antibody (#ab128568; Abcam, Cambridge, MA, USA) or β‐actin antibody (#4970; Cell Signaling Technology).

### 2.6 Liquid chromatography‐tandem mass spectrometry analysis

Gastric cancer tissues and the matching adjacent non‐tumour tissues were from seven GC patients and combined respectively. The samples were prepared and determined the protein lysine succinylation by liquid chromatography‐tandem mass spectrometry (LC‐MS/MS) analysis in PTM Bio.

### 2.7 Immunoprecipitation

Cells were harvested and lysed in immunoprecipitation (IP) buffer (20 mM Tris, pH 7.5, 150 mM NaCl, 1% Triton X‐100, 1 mM EDTA, and protease inhibitors) on ice for more than 15 minutes. Cell lysates were centrifuged for 10 minutes at 12 000 *g* at 4°C, and supernatant were transferred to new tubes. The supernatant was incubated with primary antibodies and GammaBind Plus Sepharose (#17088601; GE Healthcare, Logan, UT, USA) with gentle rocking overnight at 4°C. The next day, the pellet was washed six times with cold 1× IP buffer and then subjected to western blotting.

Frozen tissues were homogenized in ice‐cold 0.3% NP‐40 buffer containing 50 mM Tris–HCl (pH 7.4), 150 mM NaCl, and protease inhibitors. S100A10 protein was immunoprecipitated with an anti‐S100A10 antibody (sc‐81153; Santa Cruz Biotechnology, Dallas, TX, USA), followed by direct Western blot analyses as described above.

### 2.8 Plasmid construction and cell transfection

Full‐length WT cDNA or cDNA with point mutations of the *S100A10* gene was synthesized (Wuxi Qinglan Biotech. Inc., Yixing, China) and cloned into indicated vectors including pRF‐FLAG or pRF‐HA (kindly obtained from Prof. Hongbing Shu). *SIRT5* gene clone was purchased from Shanghai Genechem Co., Ltd. (Shanghai, China) and subsequently cloned into the pRF‐HA vector. Cell transfection was performed with Lipofectamine 3000 (Invitrogen).

### 2.9 In vitro desuccinylation assay

FLAG‐S100A10, HA‐tagged WT SIRT5 or a catalytic inactive mutant SIRT5 (H158Y) was overexpressed in HEK293T cells. Proteins were immunoprecipitated with anti‐Flag M2 or HA antibody and beads, and then eluted with Flag or HA peptides respectively. FLAG‐S100A10 protein was incubated with HA‐tagged wild‐type or mutant SIRT5 in reaction buffer (80 μL) containing 25 mM Tris–HCl (pH 8.0), 1 mM MgCl_2_, 200 mM NaCl, 5 mM KCl, 0.1% PEG8000, and 3.125 mM NAD^+^ at 37°C for 1 hour, and then subjected to Western blot analysis.

### 2.10 RNA interference analysis

Down‐regulation of SIRT5 was performed by RNA interference. Scrambled, human *SIRT5* shRNAs and human *CPT1A* shRNAs were obtained from Shanghai Genechem Co., Ltd. and used according to the protocols provided by the manufacturer. The cells were harvested at the indicated time‐points and were subjected to western blot analysis. All shRNA transfections were performed with Lipofectamine 3000 (Invitrogen) as described by the manufacturer. Knockdown efficiency was verified by western blotting.

### 2.11 Immunohistochemical and histological analyses

The succinylated S100A10 peptide, CFLENQK_succ_DPLAV‐NH_2_, was synthesized and used to prepare rabbit polyclonal antibody from ChinaPeptides Co., Ltd. (Shanghai, China). For immunohistochemical (IHC) staining, 5‐μm thick serial sections were used to prepare the slides. Antigen retrieval was performed with 10 mM citrate antigen retrieval solution (CW Biotech, Beijing, China) at 95°C for 10 minutes. The endogenous peroxidase activity was inactivated using endogenous peroxidase enzyme blocking buffer. After non‐specific interactions were blocked with normal goat serum, S100A10 rabbit polyclonal antibody (#HPA003340; Sigma‐Aldrich) at a dilution of 1:200 or S100A10‐K47_suc_ rabbit polyclonal antibody at a dilution of 1:500 was incubated with the slides overnight at 4°C. The following detection and visualization procedures were performed according to the manufacturer's protocol (CW Biotech, Beijing, China): for histological analysis, tissues were fixed in 10% formalin, embedded in paraffin, sectioned (4 μm), and stained with haematoxylin and eosin (H&E) for light microscopy (Olympus IX51, Tokyo, Japan).

### 2.12 Generation of stable cell lines

The stable cell lines were generated using lentivirus system. Briefly, the genes were cloned into pLJM1 vector, and then cotransfected into HEK293T cells together with pMD2.G and psPAX2 vectors using Lipofectamine 3000 reagent. Lentiviral supernatants were harvested from HEK293T cells at 48 hours after initial plasmid transfection and mixed with 8 μg/mL of polybrene to increase the infection efficiency. The infected cancer cells were then selected in culture media containg 2 μg/mL of puromycin for 2 weeks.

### 2.13 Cell invasion and wound healing assays

AGS cells (1 × 10^5^) in 200 μl serum‐free DMEM were seeded in a Transwell apparatus (Corning Life Sciences, Corning, NY, USA) pre‐coated with 60 μL Matrigel (1:3 dilution; BD Biosciences, San Jose, CA, USA). Then, cells were transfected with FLAG‐S100A10^WT^ or FLAG‐S100A10^K47E^ for 48 hours at 37°C. Cells that adhered to the lower surface were fixed with 100% methanol for 15 minutes at room temperature and subsequently stained with crystal violet for 15 minutes. In each replicate, cells were counted in six predetermined fields under a microscope. For wound healing assays, MGC‐803 cells were transfected with FLAG‐S100A10^WT^ or FLAG‐S100A10^K47E^ for 24 hours at 37°C, and then monolayer cells were wounded with a sterile plastic tip. Cell migration was observed by microscopy 16 and 24 hours later. Migration was quantified as a percentage of wound closure. The assay was repeated at least three times independently.

### 2.14 Assessment of melanoma lung metastasis

B16‐F10 cells were administered to mice by tail‐vein injection (2 × 10^5^ cells/mouse in 200 μL DMEM; n = 6 per group). Lung melanoma metastases were quantified by counting the number of colonies that appeared as black dots on the pleural surface.

### 2.15 Statistical analysis

All experiments were repeated at least three times. All values in the figures and text are expressed as mean ± SEM. Two‐tailed Student's *t*‐tests were used to compare two groups. All statistical analyses were performed with GraphPad Prism (v6.0, GraphPad Software, San Diego, CA, USA). Differences were considered significant at *P* < 0.05.

## 3 RESULTS

### 3.1 S100A10 is overexpressed in GC

**Figure 1:**
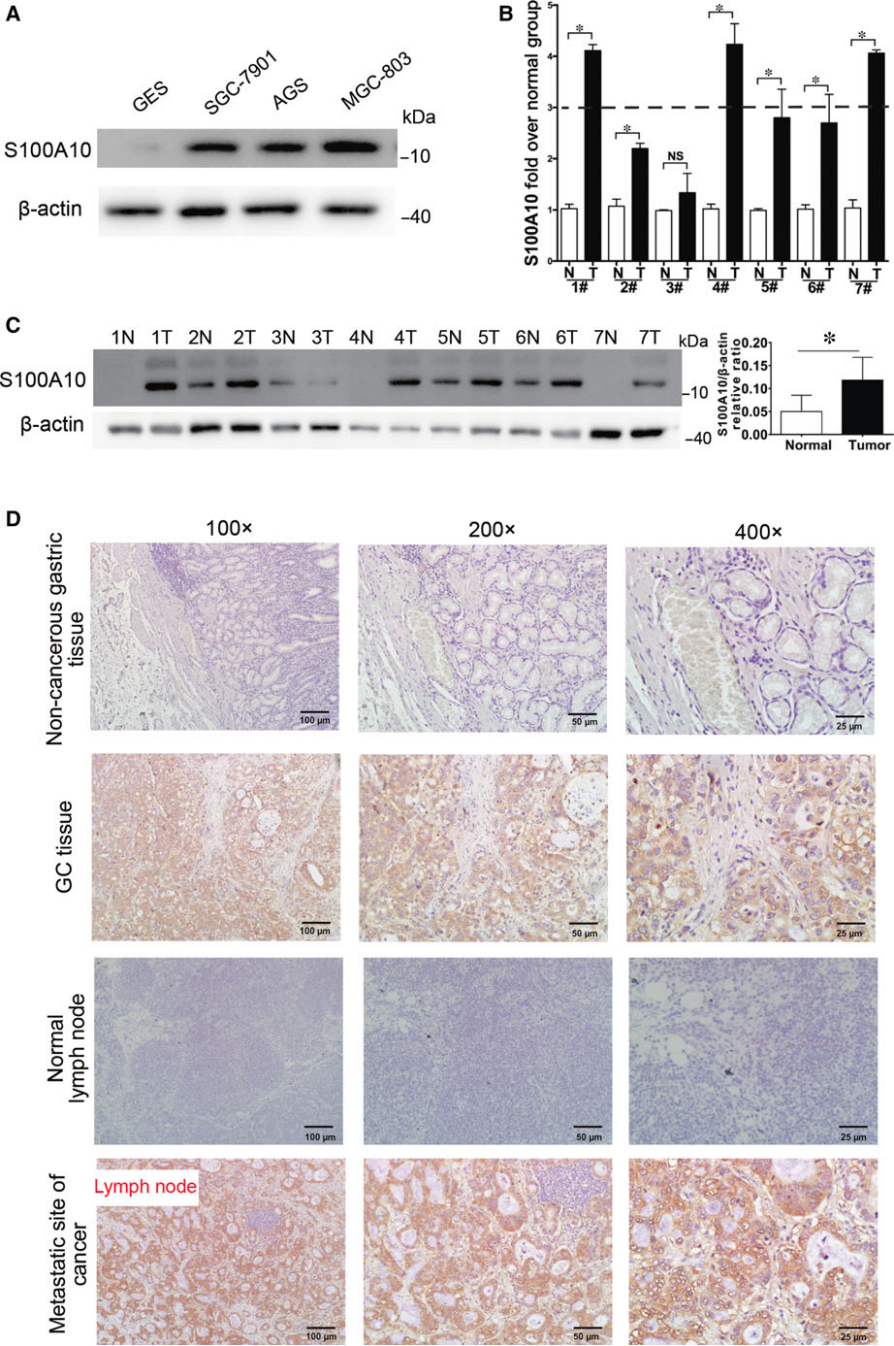
Expression of S100A10 in human gastric cancer (GC) samples. A, Western blot analysis of S100A10 protein expression in various cell lines. B, qRT‐PCR analysis of the mRNA levels of *S100A10* in seven pairs of gastric tissues (GC and adjacent non‐cancerous tissues). C, Western blot analysis of the relative levels of S100A10 protein expression in seven pairs of GC and adjacent non‐cancerous tissues. D, Expression of S100A10 detected by immunohistochemistry in non‐cancerous gastric tissues, GC tissues, adjacent non‐tumour lymph nodes, and metastatic lymph nodes of GC patients. Results are derived from ten tissue slides. Data are presented as mean ± SEM; *P < 0.05

First, we examined the expression level of S100A10 in a panel of human GC and immortalized human gastric epithelial mucosa GES‐1 cells. The results showed that S100A10 was highly expressed in MGC‐803, AGS, and SGC7901 cells when compared with levels in GES‐1 cells (Figure 1A). Next, we collected a total of seven primary GC samples and paired surrounding normal gastric mucosa samples and performed qRT‐PCR and western blotting analyses. *S100A10* mRNA levels were significantly lower in normal tissues than in cancer tissues (Figure 1B). Consistent with this, there was a significant increase in the steady‐state levels of total S100A10 protein in tumour tissues compared to levels in normal gastric tissues (Figure 1C). Furthermore, the expression of S100A10 in GC tissues, adjacent normal tissues, and in normal lymph node and metastatic lymph node tissues of GC patients were investigated by IHC. No apparent immunopositivity for S100A10 was observed in the normal gastric mucosa or normal lymph node. In contrast, GC tissues and metastatic lymph node tissues showed obvious positive staining of S100A10 (Figure 1D). Taken together, these results suggest that S100A10 is overexpressed in GC tissues and metastatic lymph nodes associated with an aggressive tumour phenotype.

### 3.2 S100A10 is succinylated at lysine 47 in GC

**Figure 2:**
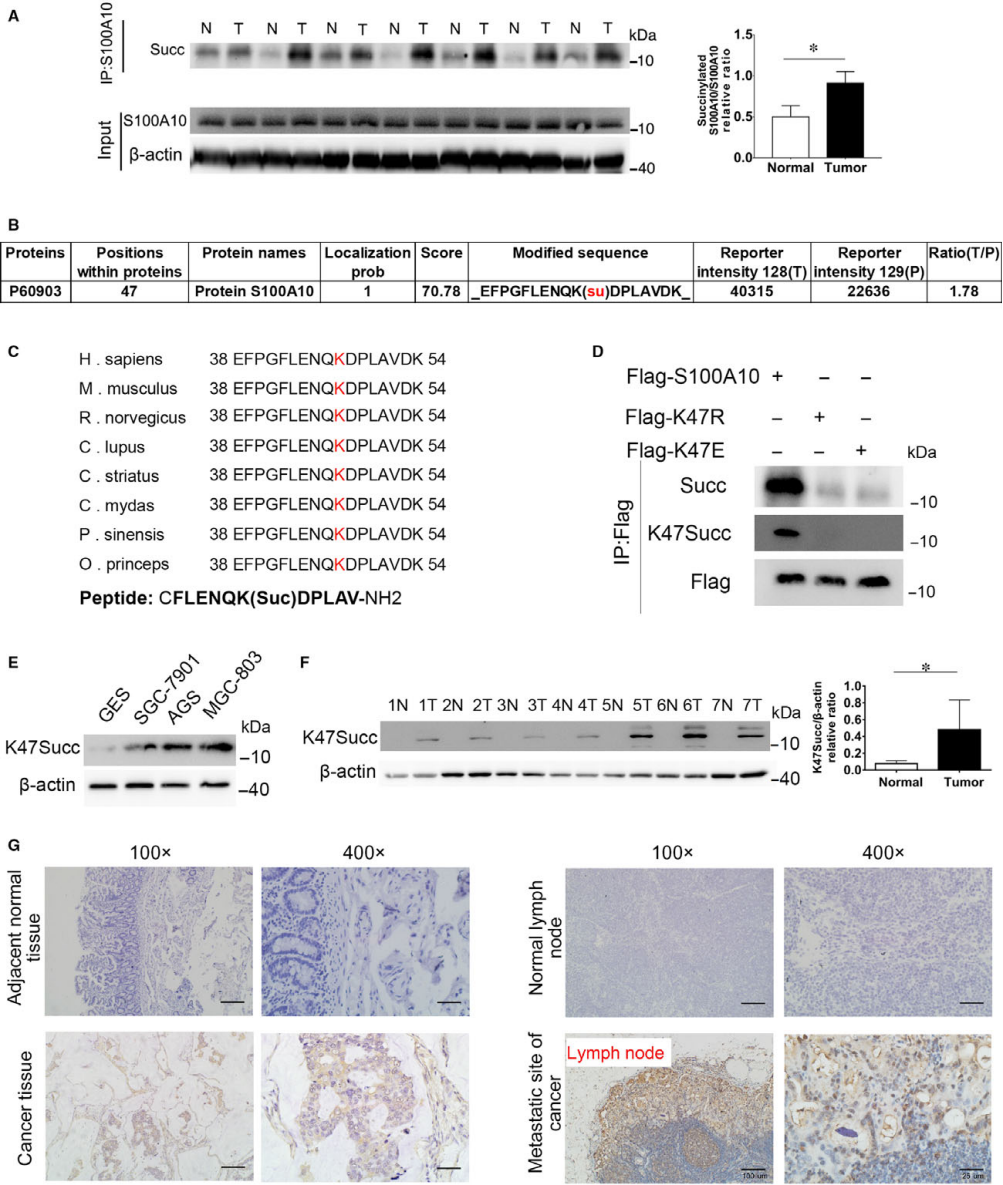
S100A10 is succinylated at lysine 47 in GC. A, Immunoprecipitation analysis of S100A10 succinylation in GC tissues. B, Identification of succinylated S100A10 peptides by mass spectrometry of GC tissues. C, Protein amino acid sequence alignment of S100A10 surrounding K47 from various organisms. K47 is shown in red. The peptide sequence used for preparing the antibody is indicated. D, K47 is the primary succinylation site of S100A10. The indicated plasmids were transfected into MGC‐803 cells and proteins were immunoprecipitated, followed by western blotting for succinylation analysis. E, Western blot analysis of S100A10 K47_succ_ expression in various cell lines. F, Western blot analysis of the relative levels of S100A10 K47_succ_ expression in seven pairs of GC and adjacent non‐cancerous tissues. G, Immunohistochemistry of S100A10 K47_succ_ in adjacent normal tissues, GC tissues, normal lymph nodes and metastatic lymph nodes of GC patients. Results are derived from ten tissue slides. Data are presented as mean ± SEM; *P < 0.05

First, we measured protein PTM including succinylation, crotonylation, and acetylation in seven pairs of gastric tissues (GC and adjacent non‐cancerous tissues) from GC patients. Our data indicated that protein modification by succinylation was more consistent than crotonylation and acetylation in GC and adjacent tissues from those patients (Figure S1A‐C). Therefore, we focus on studying protein succinylation modification in GC. Lysine succinylation is a relatively novel PTM, and its physiological significance in GC remains unclear. We immunoprecipitated S100A10 from a panel of seven pairs of primary gastric tumours and their adjacent normal tissues to investigate whether this modification was present. Pan‐succinyl‐lysine antibody was used to examine the succinylation of S100A10 in GC tissues. Results showed that lysine succinylation of S100A10 was more prevalent in tumour tissues than in normal tissues (Figure 2A). In addition, malonylation and glutarylation of S100A10 were also determined. However, no difference was observed between GC and normal tissues (Figure S1D). To determine the specific K_succ_ sites in S100A10 in GC, a total of seven tumour tissues or paired adjacent normal tissues were lysed and pooled. After trypsin digestion, the proteins were analysed by the integrated methods of TMT labelling, HPLC fractionation, affinity enrichment, and LC‐MS/MS (PTM Bio). Altogether, 503 lysine succinylation sites in 303 proteins were identified (data not shown), among which S100A10 protein was succinylated only at lysine 47 (K47) and up‐regulated 1.78 folds in GC tissues (Figure 2B). We analysed the conservation of K47 and found that this site is highly conserved in S100A10 across various species (Figure 2C).

Next, we generated a K47 site‐specific antibody using a human S100A10 peptide succinylated at K47 as an antigen. The specificity of the anti‐K47_succ_ antibody was verified by detecting a specific band with WT S100A10 but not with K47 mutants (Figure 2D). To further confirm whether S100A10 is succinylated at lysine 47, FLAG‐tagged WT, 47K‐to‐R mutant (mimicking deletion), or 47K‐to‐E mutant (mimicking the negatively charged succinyl lysine modification) S100A10 was expressed in MGC‐803 cells. As a result, both mutations decreased the overall succinylation levels of S100A10 (Figure 2D). Furthermore, levels of the K47_succ_‐S100A10 protein were significantly elevated in human GC cells when compared to those in GES‐1 cells (Figure 2E). Moreover, GC tissues exhibited higher levels of K47_succ_‐S100A10 than normal tissues according to western blotting assays (Figure 2F). IHC analysis also show that K47_succ_‐S100A10 protein was highly expressed in GC and metastatic lymph node tissues, not in normal gastric mucosa or lymph node tissues (Figure 2G).

### 3.3 S100A10K47 succinylation inhibits S100A10 degradation

**Figure 3:**
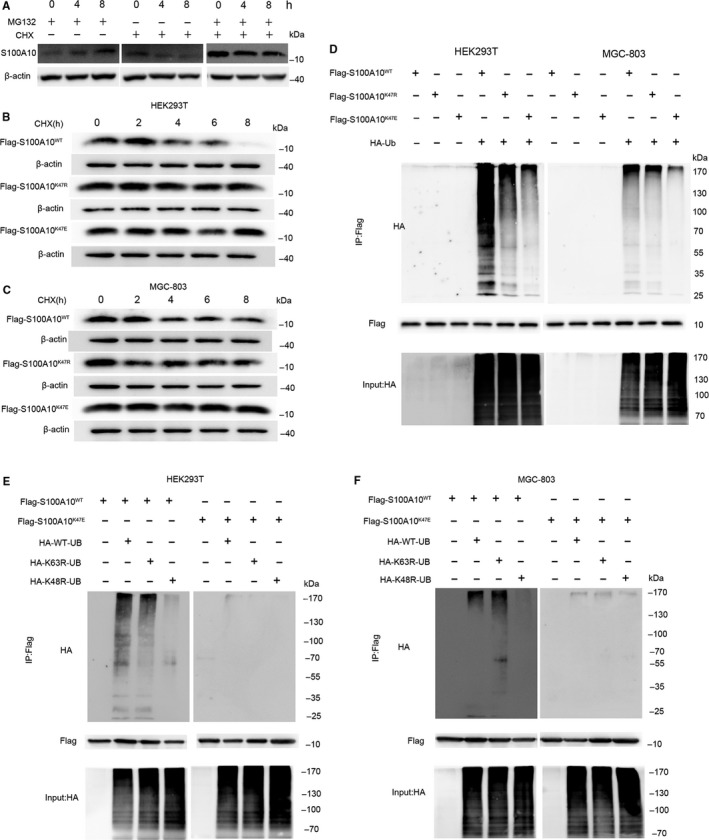
K47 succinylation stabilizes S100A10 protein. A, MG132 treatment stabilizes endogenous S100A10. MGC‐803 cells were cultured with CHX (10 μg/mL) or MG132 (10 mM), and cell lysates were analysed by western blotting. B and C, CHX treatment shortens the half‐life of WT, but not K47R or K47E mutant, S100A10. HEK293T or MGC‐803 cells expressing WT or K47R/K47E mutant S100A10 were cultured in the presence of CHX (10 μg/mL) for various durations. S100A10 protein levels were determined by western blotting. D, K47E mutation blocks S100A10 ubiquitylation in HEK293T cells and MGC‐803 cells. HEK293T cells and MGC‐803 cells were transfected with the indicated plasmids. Ubiquitylation of purified FLAG‐S100A10 protein was measured by western blotting. E and F, Either WT or K47E mutant FLAG‐S100A10 was transfected with plasmids containing no ubiquitin, HA‐WT‐ubiquitin, HA‐K63R‐ubiquitin or HA‐K48R‐ubiquitin into HEK293T cells and MGC‐803 cells. Ubiquitylation of purified FLAG‐S100A10 proteins was determined by western blotting

Unpartnered S100A10 is rather unstable, and it becomes polyubiquitinated and degraded via proteasome‐dependent proteolysis.^31^ Therefore, we next determined whether endogenous S100A10 protein in MGC‐803 cells is degraded via a proteasomal mechanism. Treatment of MGC‐803 cells with the proteasomal inhibitor MG132 resulted in increased expression of S100A10 (Figure 3A), while treatment with the protein synthesis inhibitor CHX dramatically decreased the half‐life of endogenous S100A10 (Figure 3A). In addition, WT, K47R and K47E S100A10 protein expression was determined following CHX treatment in transfected HEK293T or MGC‐803 cells. CHX dramatically decreased the amount of WT S100A10 but did not affect levels of S100A10 mutants (Figure 3B and C), indicating that proteasomal degradation of S100A10 might be related to K47 succinylation. Furthermore, we employed a ubiquitylation ladder assay in transfected HEK293T or MGC‐803 cells and observed a significant decrease in the ubiquitylation of K47E S100A10 (Figure 3D) compared to that of WT S100A10.

The roles of Lys48‐ and Lys63‐linked polyubiquitin in protein degradation and cellular signalling are well characterized.^32^ Therefore, we next determined whether the polyubiquitin chain on S100A10K47 was K63‐ or K48‐linked. Replacement of only the lysine in ubiquitin position 48 with arginine (R) prevented the ubiquitylation of WT S100A10, whereas replacement of the lysine at position 63 with arginine did not affect ubiquitylation (Figure 3E and F). Furthermore, no ubiquitylation was observed in the K47E mutant S100A10 (mimicking the succinyl lysine modification) in transfected HEK293T or MGC‐803 cells (Figure 3E and F). Collectively, these results indicate that K47 succinylation of S100A10 inhibits its ubiquitylation‐mediated proteasomal degradation.

### 3.4 SIRT5 decreases S100A10 succinylation

**Figure 4:**
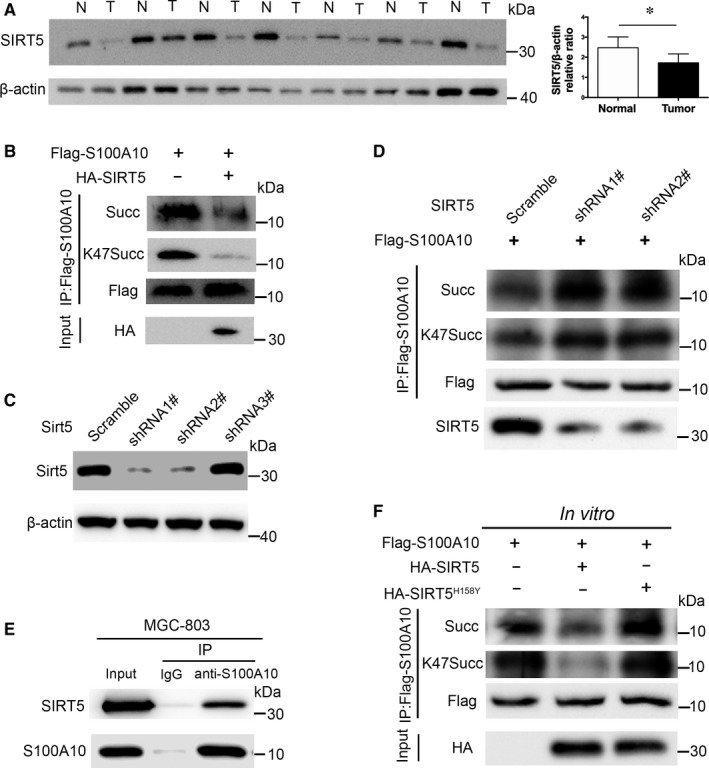
Succinylation of S100A10K47 is regulated by SIRT5. A, Western blot analysis of SIRT5 expression in GC (T) and adjacent non‐cancerous tissues (N). Data are presented as mean ± SEM; *P < 0.05. B, Overexpression of WT SIRT5 in MGC‐803 cells reduced the succinylation of S100A10. C, *SIRT5* knockdown efficiency was determined by western blotting in MGC‐803 cells. D, Knockdown of *SIRT5* enhanced the succinylation of S100A10 in FLAG‐S100A10‐transfected MGC‐803 cells. E, The interaction between endogenous SIRT5 and S100A10 in MGC‐803 cells was determined by co‐IP followed by western blotting. F, SIRT5 dessucinylates S100A10 in vitro. FLAG‐S100A10, HA‐tagged wild‐type SIRT5 or a catalytic inactive mutant SIRT5 (H158Y) was overexpressed in HEK293T cells and immunoprecipitated by FLAG or HA antibody. After incubation with wild‐type or mutant SIRT5 in vitro, the succinylation level of S100A10 protein was determined by Western blot analysis

Lysine succinylation is commonly regulated by the deacylase SIRT5.^17^, ^33^ Therefore, we determined the expression of SIRT5 in GC tissues. Intriguingly, SIRT5 protein levels were significantly reduced in GC tissues compared to those in the adjacent normal tissues (Figure 4A), indicating that the SIRT5 desuccinylase may be involved in S100A10K47 desuccinylation. Therefore, we examined the role of *SIRT5* overexpression or knockdown in K47_succ_‐S100A10 expression. The overexpression of *SIRT5* dramatically reduced K47_succ_‐S100A10 levels (Figure 4B). To deplete human *SIRT5* levels, three shRNA sequences were generated, and shSIRT5‐1# and 2# were shown to be more potent (Figure 4C). Consequently, knockdown of *SIRT5* with these two shRNAs significantly increased the succinylation level of S100A10 at K47 (Figure 4D). Moreover, the interaction between endogenous SIRT5 and S100A10 in MGC‐803 cells was demonstrated by co‐IP followed by western blotting (Figure 4E). Furthermore, we found that WT SIRT5, but not a catalytic inactive mutant SIRT5 (H158Y) mutant, could reduce the succinylation level of FLAG‐S100A10 in vitro (Figure 4F). Taken together, our data demonstrate that S100A10 is succinylated at K47 and is regulated by the SIRT5 desuccinylase.

### 3.5 CPT1A binds to and succinylates S100A10 at K47

**Figure 5:**
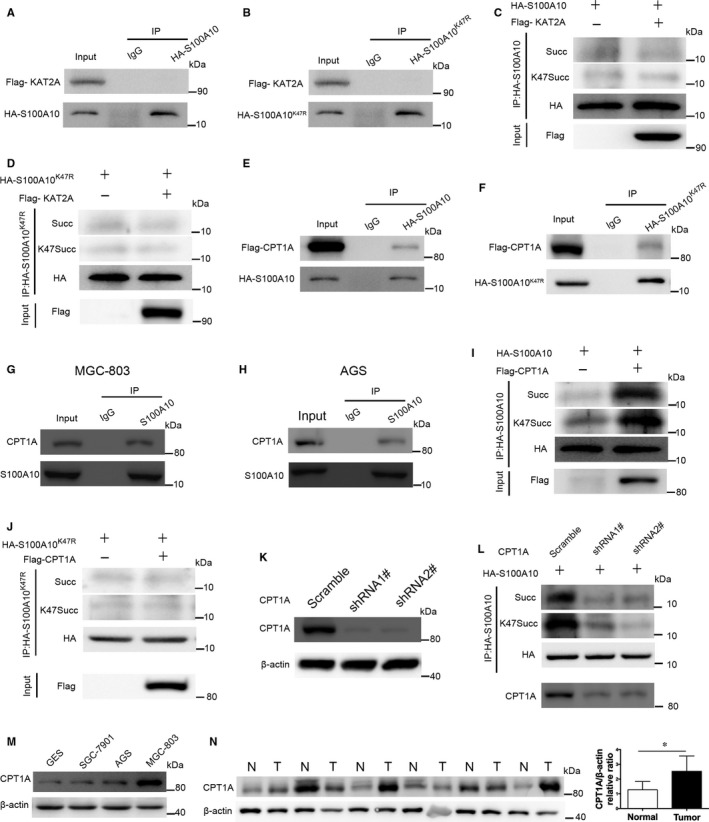
CPT1A, not KAT2A, binds to and succinylates S100A10 at K47. A and B, KAT2A did not bind to S100A10. HEK293T cells were transfected with KAT2A and S100A10 (A) or S100A10 K47R mutant (B). The binding of S100A10 or its mutant to KAT2A was examined by co‐IP and western blot. C and D, Overexpression of WT KAT2A in MGC‐803 cells did not increase the succinylation of S100A10 (C) or S100A10 K47R mutant (D). E‐H, CPT1A binds to S100A10. HEK293T cells were transfected with CPT1A and S100A10 (E) or S100A10 K47R mutant (F). The binding of S100A10 or its mutant to CPT1A was determined by co‐IP and western blot. In addition, the interaction between endogenous CPT1A and S100A10 in MGC‐803 (G) or AGS cells (H) was determined by co‐IP followed by western blotting. I and J, Overexpression of CPT1A in HEK293T cells increased the succinylation of S100A10 (I), not K47R mutant (J). K, *CPT1A* knockdown efficiency was determined by western blotting in MGC‐803 cells. L, Knockdown of *CPT1A* decreased the succinylation of S100A10 in FLAG‐S100A10‐transfected MGC‐803 cells. M, Western blot analysis of CPT1A protein expression in various GC cell lines. N, Western blot analysis of CPT1A expression in GC (T) and adjacent non‐cancerous tissues (N). Data are presented as mean ± SEM; *P < 0.05

Since the histone acetyltransferase KAT2A can succinylate nuclear histone H3,^30^ we determined whether KAT2A binds to and succinylates S100A10. However, KAT2A did not interact with or succinylate S100A10 (Figure 5A and C) or K47R mutant (Figure 5B and D). CPT1A has also been shown to have lysine succinyltransferase activities.^29^ Intriguingly, co‐IP assay indicated that exogenous CPT1A could bind to S100A10 or K47R mutant in HEK293T cells (Figure 5E and F). Furthermore, the interaction between endogenous CPT1A and S100A10 in MGC‐803 (Figure 5G) and AGS cells (Figure 5H) was demonstrated by co‐IP followed by western blotting. Moreover, overexpression of CPT1A increased the K47 succinylation of S100A10 (Figure 5I), not K47R mutant (Figure 5J). Accordingly, knockdown of CPT1A decreased the succinylation of S100A10 in FLAG‐S100A10‐transfected MGC‐803 cells (Figure 5L). We further examined CPT1A levels in human GC. Notably, CPT1A expression in human GC cells was significantly higher than that in GES‐1 cells (Figure 5M). Accordingly, GC tissues exhibited a significant increase in the expression of CPT1A compared with that in normal tissues (Figure 5N).

### 3.6 K47 succinylation enhances the function of S100A10 in supporting metastasis

**Figure 6:**
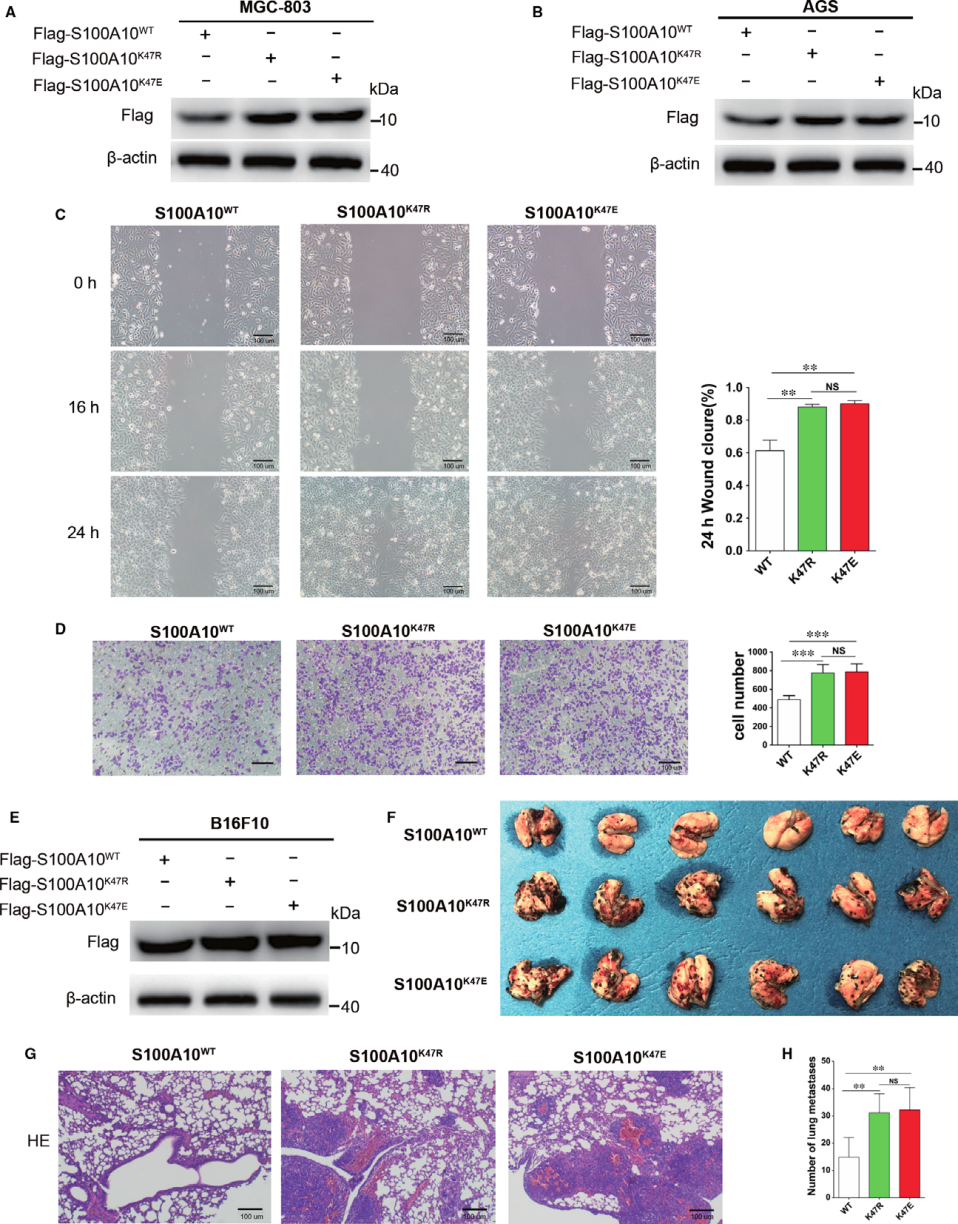
Succinylation mimetic S100A10 K47E mutant enhances cancer cell invasion and migration. A and B, Overexpression of WT, K47R, or K47E mutants were determined by western blotting in MGC‐803 (A) and AGS cells (B). C, S100A10 K47R or K47E mutant promotes cell migration. The migration of transfected MGC‐803 cells was analysed by wound‐healing assay. Scale bars represent 100 μm. D, The effect of S100A10 WT, K47R or K47E on AGS cell invasion was determined. E, Overexpression of WT or K47R/E mutants in B16F10 cells was determined by western blotting. F, Mouse model of B6F10 lung metastasis, visible as black dots on the surface of lungs from three groups. G, Representative H&E sections of lung tissues from each group. H, Total numbers of melanoma lung metastatic colonies observed in these groups. Data shown are representative of three independent experiments. Scale bars represent 100 μm. Date are presented as mean ± SEM; **P < 0.01, ***P < 0.001

S100A10 is essential for cancer cell invasion and migration. Therefore, we investigated the effect of S100A10K47 succinylation on GC cell progression. WT, K47R or K47E mutant S100A10 was overexpressed in MGC‐803 or AGS cells (Figure 6A and B). As determined by wound‐healing assays, the S100A10K47R or K47E mutation increased MGC‐803 cell migration compared with that observed with WT S100A10 (Figure 6C). Similarly, the invasion of AGS cells was promoted by the S100A10 K47R or K47E mutation according to Transwell assays (Figure 6D), with an increased number of cancer cells passing through the Transwell membrane filter in the K47R or K47E S100A10 group (Figure 6D).

To address the biological significance of K47 succinylation in vivo, we performed mouse melanoma lung metastasis experiments using stable B16F10 cell lines overexpressing WT, K47R or K47E mutant S100A10 (Figure 6E). The expression of CPT1A protein and the level of S100A10 succinylation were much lower in B16F10 cells when compared to those in AGS and MGC‐803 cells (Figure S2). Our data show that K47R or K47E mutant S100A10‐overexpressing B16F10 cells caused more lung metastasis than WT S100A10‐overexpressing cells (Figure 6F‐H). Taken together, these data indicate that S100A10 K47 succinylation promotes tumour cell invasion and metastasis.

## 4 DISCUSSION

The S100 family of calcium‐binding cytosolic proteins, comprising 25 known members,^34^, ^35^ has been postulated to regulate various cellular processes, including channel dynamics, cytoskeletal assembly, calcium balance, cell apoptosis, migration, proliferation, differentiation, inflammation and energy metabolism.^21^, ^36^, ^37^, ^38^, ^39^ Among these, S100A10 is widely expressed in numerous different types of human cancer, including lung adenocarcinoma, ovarian cancer and colorectal cancer.^9^, ^10^, ^11^, ^40^, ^41^ In this study, we further demonstrated that S100A10 is overexpressed in human GC tissues. As a plasminogen receptor, S100A10 is thought to play an important role in tumour invasiveness, angiogenesis, and tumour metastasis.^42^, ^43^, ^44^ Consistent with this, we found strong S100A10 expression in metastatic lymph nodes of GC patients, suggesting that S100A10 may promote GC metastasis. However, the exact molecular mechanism regulating S100A10 expression in GC remains unknown. Its widespread up‐regulation in cancer is in part due to transcriptional activation.^12^ Alone, the S100A10 protein is unstable, and its association with annexin A2 protects S100A10 from ubiquitin‐dependent proteolysis.^31^ This also suggests that the regulation of S100A10 at the protein level may be more common than its regulation at the gene level.

Ubiquitylation plays a crucial role in S100A10 degradation, and its binding partner, annexin A2, regulates its polyubiquitination and degradation.^31^, ^45^ In fact, mass spectrometry data from other studies have indicated that K47 of S100A10 is able to be ubiquitylated.^46^, ^47^ Our current results show that S100A10 is succinylated at K47, and this modification is more prevalent in GC tissues than in normal tissues. Succinylation, a conserved type of lysine PTM, was recently identified in many eukaryotic organisms and found to be widespread among mitochondrial, cytosolic, and nuclear proteins.^28^, ^33^ Like phosphorylation, succinylation of lysine residues also changes the charge status from +1 to −1 under physiological conditions, whereas lysine acetylation changes the charge on lysine from +1 to 0. In addition, succinylation adds a four‐carbon acyl group to lysine, while acetylation adds two carbons.^19^ Taking into account the effect of protein phosphorylation and acetylation on cell function,^28^, ^29^ lysine succinylation, overlapping extensively with acetylation,^27^ could also exert an influence on the functions of proteins and cells. Here, we found that S100A10 undergoes a high degree of succinylation in GC. Moreover, the K47E mutation (mimicking the succinyl lysine modification) appears to interfere with the ubiquitylation of S100A10. Our data also demonstrated that K48 but not K63 polyubiquitin chains link to S100A10. Therefore, we postulate that K47 succinylation stabilizes S100A10 by inhibiting its ubiquitylation and subsequent proteasomal degradation, which enhances plasminogen activation and promotes tumour cell invasion and migration.

We next sought to determine the specific molecular mechanism by which S100A10 succinylation is regulated. First, we investigated which desuccinylases could act on S100A10. The deacylase SIRT5 is known to exert desuccinylation activity.^17^, ^33^ Here, we demonstrated that overexpression of SIRT5 attenuated the K47 succinylation of S100A10, while knockdown of SIRT5 enhanced its succinylation. Moreover, Endogenous SIRT5 bound to S100A10 protein in GC cells. Notably, WT SIRT5, not a catalytic inactive mutant SIRT5 (H158Y) reduced the succinylation level of S100A10 in vitro. Second, we sought to determine which succinyltransferases could regulate S100A10. The number of known succinyltransferases is extremely limited. Recently, however, the acetyltransferases KAT2A and CPT1A were shown to exert succinylation activity.^29^, ^35^ Intriguingly, our data showed that CPT1A was highly expressed in human GC tissues and cells. Notably, we demonstrated that CPT1A rather than KAT2A bound to S100A10 and increased its K47 succinylation. Consequently, our study reveals a novel mechanism of the regulation of S100A10 ubiquitin‐dependent degradation via succinylation and provides new mechanistic insight into the upregulation of S100A10 in GC. Moreover, the fact that the stabilization of S100A10 with the K47E mutation significantly promoted cancer cell invasion in vitro and in vivo indicates the crucial role of S100A10 succinylation. It also suggests that the inhibition of S100A10 succinylation may represent a potential target for GC therapy.

In summary, our study demonstrated that S100A10 is significantly up‐regulated in human GC. This up‐regulation is enhanced by K47 succinylation, which reduces its ubiquitylation and subsequent proteasomal degradation. Moreover, we found that S100A10 succinylation was regulated by the desuccinylase SIRT5. Notably, CPT1A functions as a succinyltransferase by binding to S100A10 and promoting its succinylation, which enhances cancer metastasis and invasion. Our results may expedite the recognition of K47‐succinylated S100A10 as a novel biomarker and therapeutic target for GC.

## CONFLICT OF INTEREST

The authors declare that they have no competing interests.

## AUTHOR CONTRIBUTION

CW, CZ, XL, HW and QY designed the experiments; CW, CZ and XL performed the research; CW, CZ, XL, JS, YX, HS, XM, JP, TZ, ML, BG and CX analysed the data; CW and QY wrote the manuscript.
