# Supplementary figures and images for: CPT1A‐mediated succinylation of S100A10 increases human gastric cancer invasion
Source: J Cell Mol Med. 2018 Nov 5;23(1):293–305. doi: 10.1111/jcmm.13920 (PMC6307794; doi:10.1111/jcmm.13920)

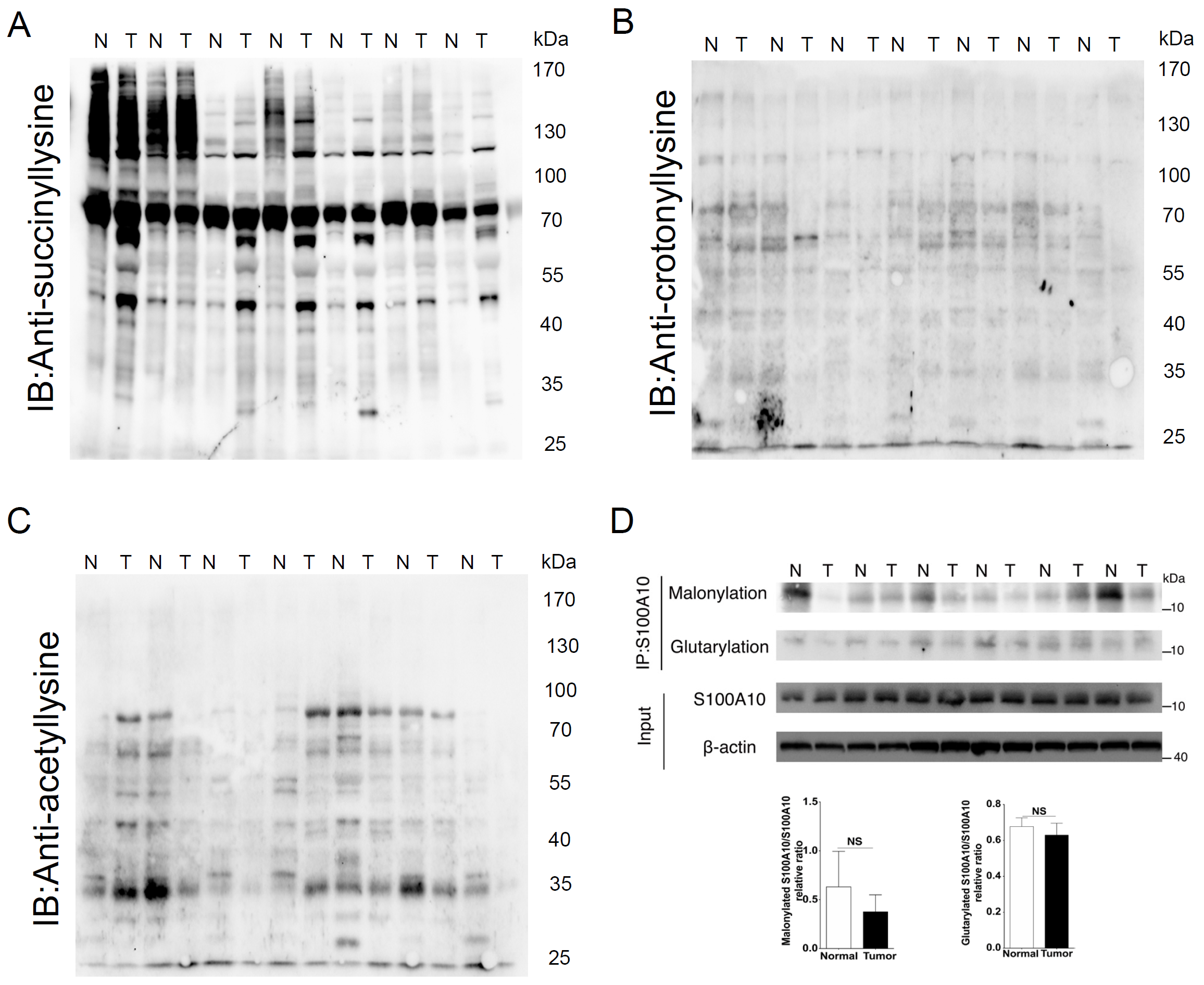

Supplement: Supplementary file 1 [file JCMM-23-293-s001.tif]

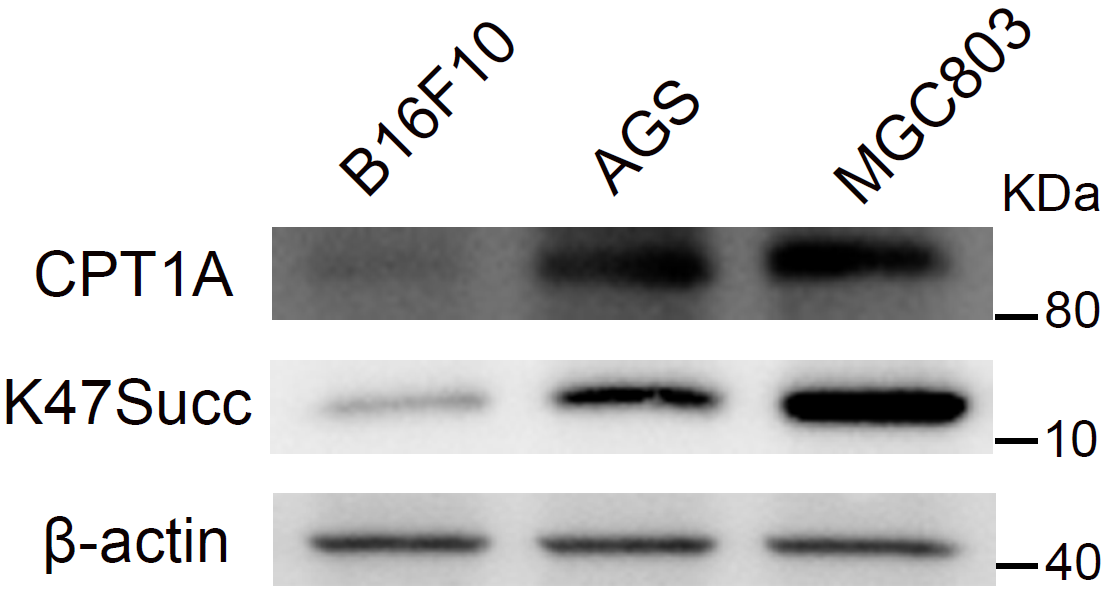

Supplement: Supplementary file 2 [file JCMM-23-293-s002.tif]
