# Supplementary material for: CPT1A‐mediated succinylation of S100A10 increases human gastric cancer invasion
Source: J Cell Mol Med. 2018 Nov 5;23(1):293–305. doi: 10.1111/jcmm.13920 (PMC6307794; doi:10.1111/jcmm.13920)
Supplement: Supplementary file 3 [file JCMM-23-293-s003.doc]

**SUPPLEMENTARY FIGURE LEGENDS**

**FIGURE S1** A-C, Protein lysine succinylation (A), crotonylation (B), and acetylation (C) were determined in seven pairs of gastric tissues (GC and adjacent non-cancerous tissues) by western blotting analysis. D, Malonylation and glutarylation of S100A10 were determined in six pairs of gastric tissues (GC and adjacent non-cancerous tissues) by co-IP followed by western blotting.

**FIGURE S2** The expression of CPT1A protein and the level of S100A10 K47 succinylation were determined in B16F10, AGS, and MGC-803 cells by western blot analysis.
